# Hexarelin alleviates apoptosis on ischemic acute kidney injury via MDM2/p53 pathway

**DOI:** 10.1186/s40001-023-01318-w

**Published:** 2023-09-14

**Authors:** Chen Guan, Chenyu Li, Xuefei Shen, Chengyu Yang, Zengying Liu, Ningxin Zhang, Lingyu Xu, Long Zhao, Bin Zhou, Xiaofei Man, Congjuan Luo, Hong Luan, Lin Che, Yanfei Wang, Yan Xu

**Affiliations:** 1 Department of Nephrology, the Affiliated Hospital of Qingdao University, 16 Jiangsu Road, Qingdao, 266003 China; 2 Medizinische Klinik Und Poliklinik IV, Klinikum Der Universität, LMU München, Munich, Germany

**Keywords:** Acute kidney injury, Kidney ischemia–reperfusion injury, Hexarelin, Apoptosis, MDM2/p53

## Abstract

**Introduction:**

Hexarelin exhibits significant protection against organ injury in models of ischemia/reperfusion (I/R)-induced injury (IRI). Nevertheless, the impact of Hexarelin on acute kidney injury (AKI) and its underlying mechanism remains unclear. In this study, we investigated the therapeutic potential of Hexarelin in I/R-induced AKI and elucidated its molecular mechanisms.

**Methods:**

We assessed the protective effects of Hexarelin through both in vivo and in vitro experiments. In the I/R-induced AKI model, rats were pretreated with Hexarelin at 100 μg/kg/d for 7 days before being sacrificed 24 h post-IRI. Subsequently, kidney function, histology, and apoptosis were assessed. In vitro, hypoxia/reoxygenation (H/R)-induced HK-2 cell model was used to investigate the impact of Hexarelin on apoptosis in HK-2 cells. Then, we employed molecular docking using a pharmmapper server and autodock software to identify potential target proteins of Hexarelin.

**Results:**

In this study, rats subjected to I/R developed severe kidney injury characterized by tubular necrosis, tubular dilatation, increased serum creatinine levels, and cell apoptosis. However, pretreatment with Hexarelin exhibited a protective effect by mitigating post-ischemic kidney pathological changes, improving renal function, and inhibiting apoptosis. This was achieved through the downregulation of conventional apoptosis-related genes, such as Caspase-3, Bax and Bad, and the upregulation of the anti-apoptotic protein Bcl-2. Consistent with the in vivo results, Hexarelin also reduced cell apoptosis in post-H/R HK-2 cells. Furthermore, our analysis using GSEA confirmed the essential role of the apoptosis pathway in I/R-induced AKI. Molecular docking revealed a strong binding affinity between Hexarelin and MDM2, suggesting the potential mechanism of Hexarelin’s anti-apoptosis effect at least partially through its interaction with MDM2, a well-known negative regulator of apoptosis-related protein that of p53. To validate these findings, we evaluated the relative expression of MDM2 and p53 in I/R-induced AKI with or without Hexarelin pre-administration and observed a significant suppression of MDM2 and p53 by Hexarelin in both in vivo and in vitro experiments.

**Conclusion:**

Collectively, Hexarelin was identified as a promising medication in protecting apoptosis against I/R-induced AKI.

**Supplementary Information:**

The online version contains supplementary material available at 10.1186/s40001-023-01318-w.

## Introduction

Acute kidney injury (AKI) is a complex syndrome marked by a decline in renal function, and it is associated with a mortality rate of up to 50% [1]. Emerging evidence suggests that AKI is not merely a self-limited condition, as recent clinical and basic research has uncovered a significant link between AKI and chronic kidney disease (CKD) [2]. Despite an extensive understanding of the pathophysiological mechanisms underlying AKI, there remains a pressing medical necessity to identify potential targeted interventions for this condition [3].

Renal ischemia–reperfusion injury (IRI) is the most common cause of AKI [4]. Given the high metabolic activity and limited oxygen supply from the capillary network at baseline [5], proximal tubules are vulnerable to ischemia damage, resulting in ATP depletion, the accumulation of reactive oxygen species, and cell apoptosis or necrosis [6, 7]. In the context of AKI, these tubules can be subjected to multiple insults, leading to cell death through apoptosis, prominent programmed and unprogrammed necrosis, and loss of the tubule structure [8–10]. Mechanically, renal tubule cell necrosis and apoptosis, along with the detachment and shedding of viable epithelial cells into the tubular lumen, contribute to the denudation of areas of the S3 areas, as well as the regulation of apoptotic genes including caspases and Bcl-2 family proteins [9, 11]. Evidence supports the involvement of p53 in mediating tubular cell injury and death in AKI, with even low to moderate levels of “pre-activation” significantly exacerbating tubular damage during AKI. In contrast, targeted ablation of p53 from renal proximal tubules results in the suppression of renal fibrosis [12].

Chemical or naturally derived small molecules have been utilized to address unmet medical needs for renal diseases [13–16]. Accumulating evidence suggests the use of various drugs, including chemical agents, natural products and combined therapies, for the treatment of IRI-mediated renal diseases, such as AKI, CKD and AKI-to-CKD [17–19]. Hexarelin, also known as Examorelin, is a synthetic analog of growth hormone releasing peptide 6 (GHRP-6) that exhibits chemically stable and potent functional properties in stimulating growth hormone release [20]. Recently, novel therapeutic effects of Hexarelin, beyond growth hormone release, have been demonstrated in studies investigating cardiac and neural IRI [21]. Studies have shown that Hexarelin counteracts H_2_O_2_-induced injury by enhancing cell viability and reducing the release of NO_2_- [22]. Furthermore, Hexarelin protects cardiac morphology and function against IRI by suppressing cardiomyocyte apoptosis in heart failure rats [23]. However, the extent to which Hexarelin protects against kidney injury, one of the most vulnerable organs to IRI, and the underlying mechanism of its action remain elusive.

The present study aims to investigate the potential protective effects of Hexarelin on I/R-induced AKI through a combination of in vivo and in vitro experiments. Additionally, the study aims to explore the underlying mechanisms using bioinformatics approaches, and finally validate the predicted mechanism using rats and cell models. By shedding light on the role of Hexarelin in apoptosis and AKI, our research provides a new perspective that can contribute to the development of strategies for AKI prevention.

## Results

### Hexarelin protects renal function and attenuates the histological lesions from IRI.

**Fig. 1:**
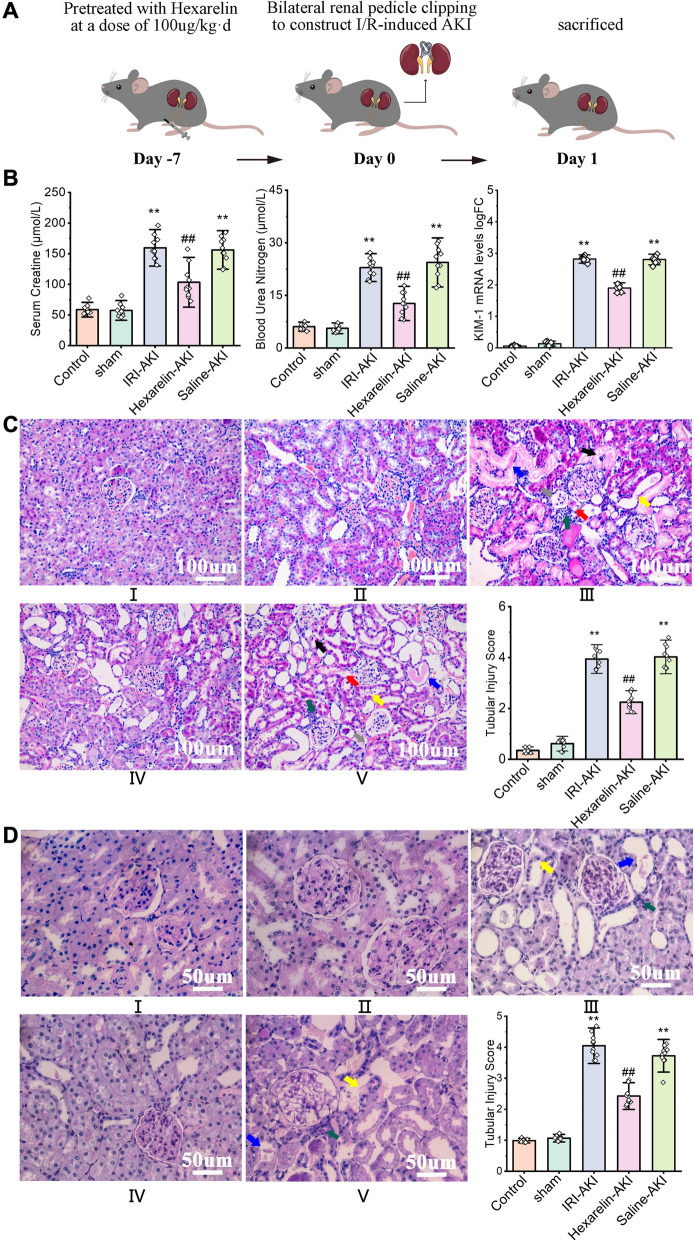
Hexarelin Attenuated Renal Function in I/R-induced AKI. **A** I/R-induced AKI model was established in SD rats; **B** serum creatine, BUN levels and relative expression of KIM-1 of different groups (**C**) H&E staining and (**D**) PAS staining of (I) control, (II) sham, (III) AKI, (IV) Hexarelin pretreatment before AKI and (V) Saline pretreatment before AKI groups; (VI) tubular injury score of different in vivo groups. Light microscopic images showing renal tubular epithelial cells shedding, tubular dilatation [yellow (III and V)], renal tubular epithelial cell necrosis, intratubular cast and basement membrane exposed [blue (III and V)], enlargement of bowman space [grey (III and V)], renal interstitial edema [red (III and V)], renal interstitial inflammatory cell infiltration [green (III and V)], severe degeneration and vacuolar of tubular epithelial cells [black (III and V)] in AKI and saline pretreatment before AKI groups. All data are shown as mean ± SD. *P < 0.05, **P < 0.01 versus control; ^#^*P* < 0.05, ^##^*P* < 0.01 versus AKI

We employed a kidney I/R rat model to investigate the impact of Hexarelin on AKI (Fig. 1A). Figure 1B demonstrates a notable increase in serum creatinine, BUN, and KIM-1 levels in the AKI group, which significantly decreased following pre-administration of Hexarelin (*P* < 0.05). The pathological changes were evaluated through semiquantitative analysis of tubular morphology by H&E and PAS staining. In Fig. 1C, both the control and sham kidneys showed normal glomerular, tubular, and vascular architecture. In contrast, the post-ischemic kidneys suffered from kidney damage, characterized by pathologic degeneration, vacuolar necrosis and shedding of renal tubular epithelial cells, tubular dilatation, intratubular cast formation, exposure of the basement membrane, enlargement of Bowman's space, renal interstitial edema, and infiltration of inflammatory cells. Notably, Hexarelin pretreatment resulted in a significant alleviation of post-ischemic kidney injury. These results were consistent with PAS staining (Fig. 1D). Taken together, treatment with Hexarelin preserved renal function after renal I/R-induced AKI.

### Hexarelin alleviates renal cell apoptosis induced by IRI

**Fig. 2:**
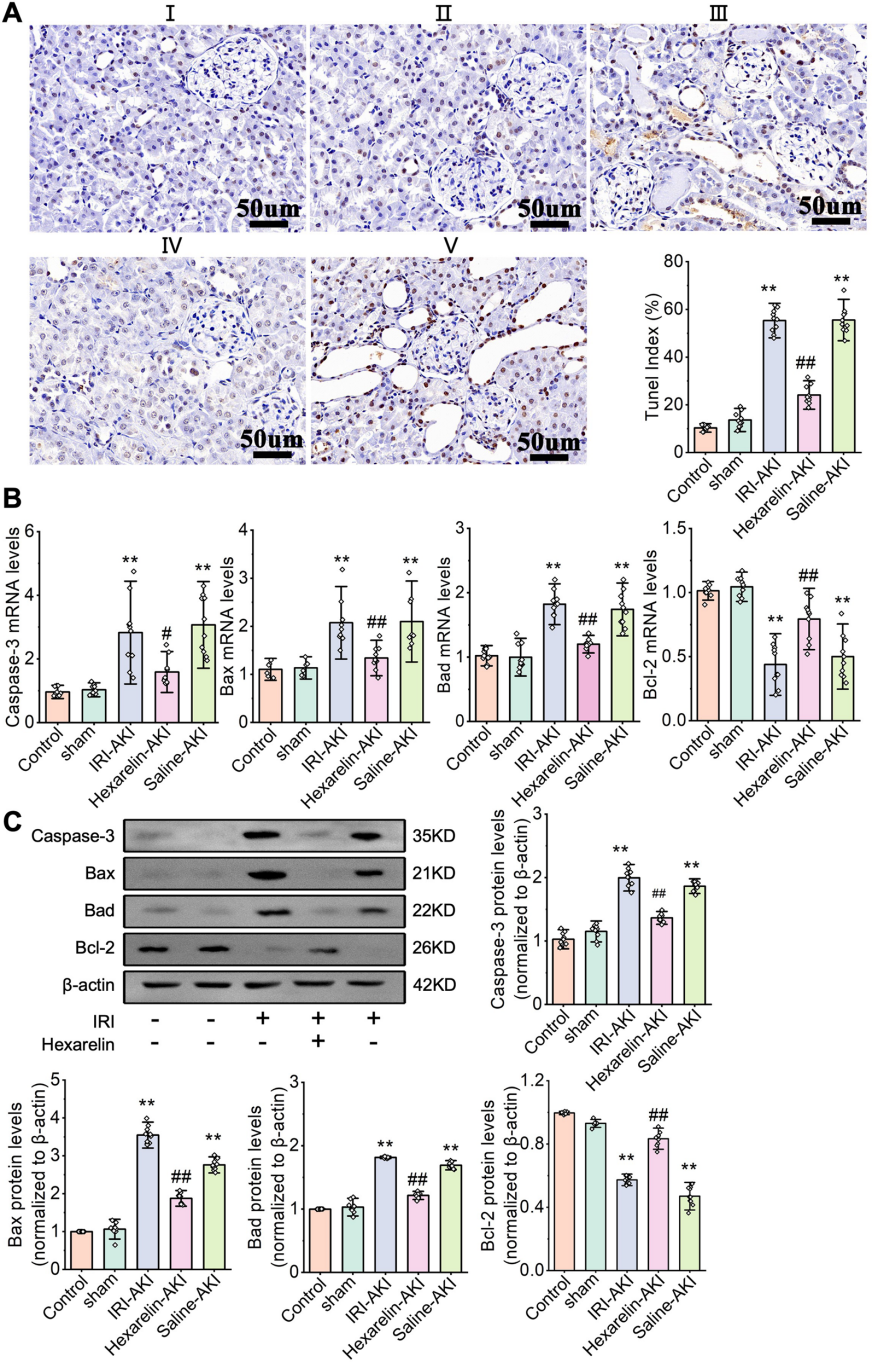
Hexarelin alleviated apoptosis after I/R-induced AKI. **A** TUNEL staining of (I) control, (II) sham, (III) AKI, (IV) Hexarelin-AKI and (V) Saline-AKI groups; (VI) quantitative analysis of TUNEL positive cell; **B**–**E** mRNA level of Caspase-3, Bax, Bad and Bcl-2; **F**–**J** Protein level of Caspase-3, Bax, Bad, Bcl-2 relative to β-actin detected by Western blotting. *P < 0.05, **P < 0.01 versus control; ^#^*P* < 0.05, ^##^*P* < 0.01 versus AKI

Apoptosis is believed to play a significant role in the pathogenesis of I/R, and the modulation of apoptosis in renal I/R holds promise for the prevention and treatment of I/R. Figure 2A illustrates the results of the TUNEL assay, as a significant increase in apoptotic cells in rats with IRI of the kidney, whereas the Hexarelin-AKI group showed a decrease in apoptotic cells (*P* < 0.05). Additionally, apoptosis-related proteins, including Caspase-3, Bax and Bcl-2, were downregulated (*P* < 0.05), while the anti-apoptotic Bcl-2 was upregulated at both mRNA and protein levels compared to the untreated post-ischemic kidney (*P* < 0.05, Fig. 2B, C). Collectively, our data indicate that Hexarelin suppresses the IRI-induced renal tubular cell apoptosis.

### Hexarelin mitigates H/R-induced cellular injury

**Fig. 3:**
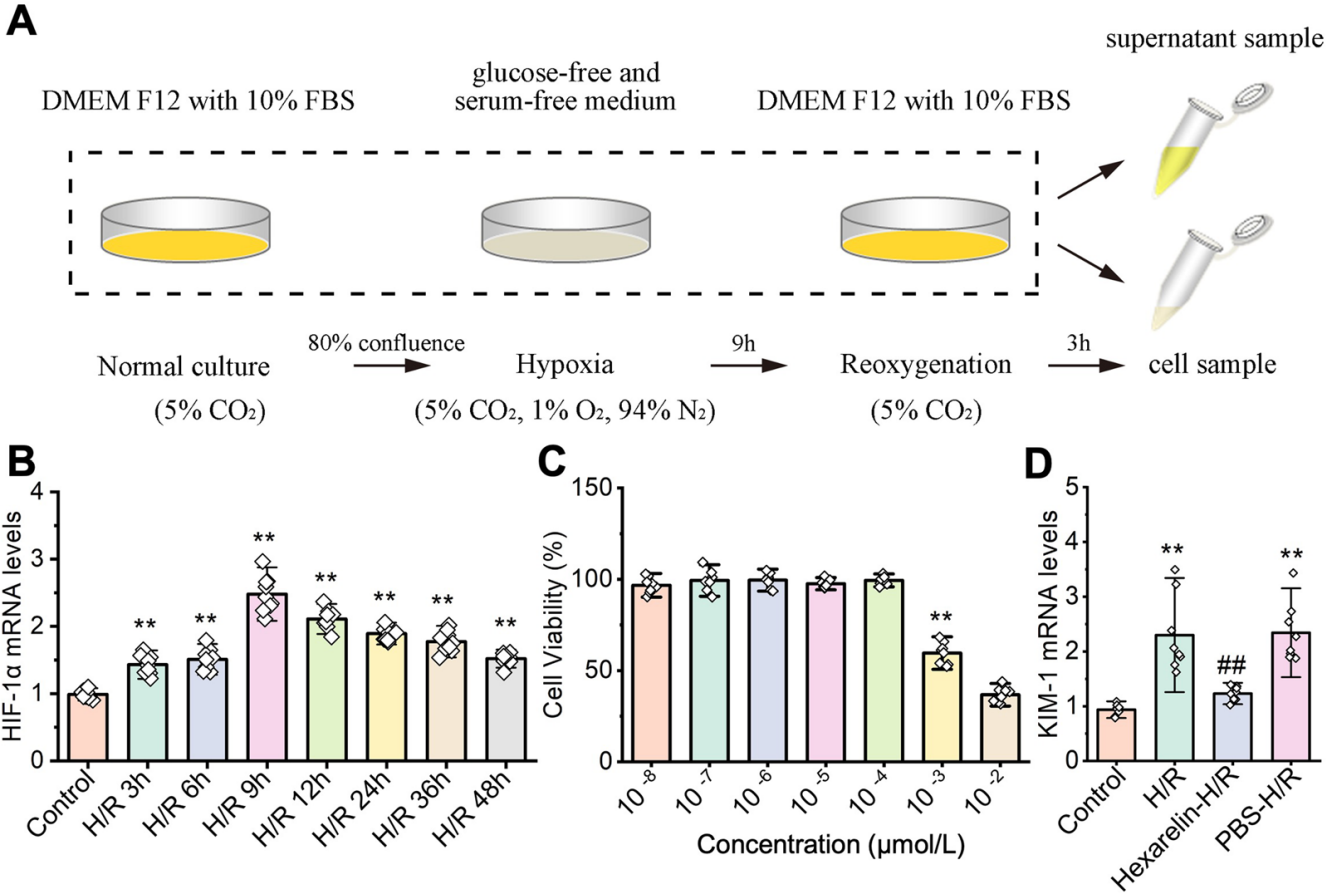
Hexarelin mitigates cell damage after H/R exposure. **A** In vitro experiments were conducted using HK-2 cells administrated with different concentrations of Hexarelin before hypoxia for 9 h followed by Reoxygenation for 3 h; **B** qRT-PCR used to measure the relative expression of Hif-1α; **C** cell viability was detected using CCK-8 assay; **D** mRNA level of KIM-1 after Hexarelin administration followed by H/R treatment. *P < 0.05, **P < 0.01 versus control; ^#^*P* < 0.05, ^##^*P* < 0.01 versus AKI

The effect of Hexarelin was further investigated in vitro using HK-2 cells exposed to various concentrations of Hexarelin incubation followed by H/R exposure (Fig. 3A). H/R induction increases the hypoxia-inducible factor 1α (HIF-1α) level, in a time-dependent way, peaking at 9 h (*P* < 0.05), which was selected for further experiments setting (Fig. 3B). The CCK-8 assay revealed that Hexarelin had no cytotoxicity with a concentration lower than 10^–4^ μmol/L (*P* < 0.05, Fig. 3C). Furthermore, Hexarelin at a dose of 10^–4^ μmol/L resulted in a significant decrease in the expression of kidney injury molecule-1 (KIM-1) post-H/R, indicating the potential protective effect of Hexarelin on H/R-induced HK-2 cells (*P* < 0.05, Fig. 3D).

### Hexarelin attenuated H/R-induced cell apoptosis in HK-2 cell

**Fig. 4:**
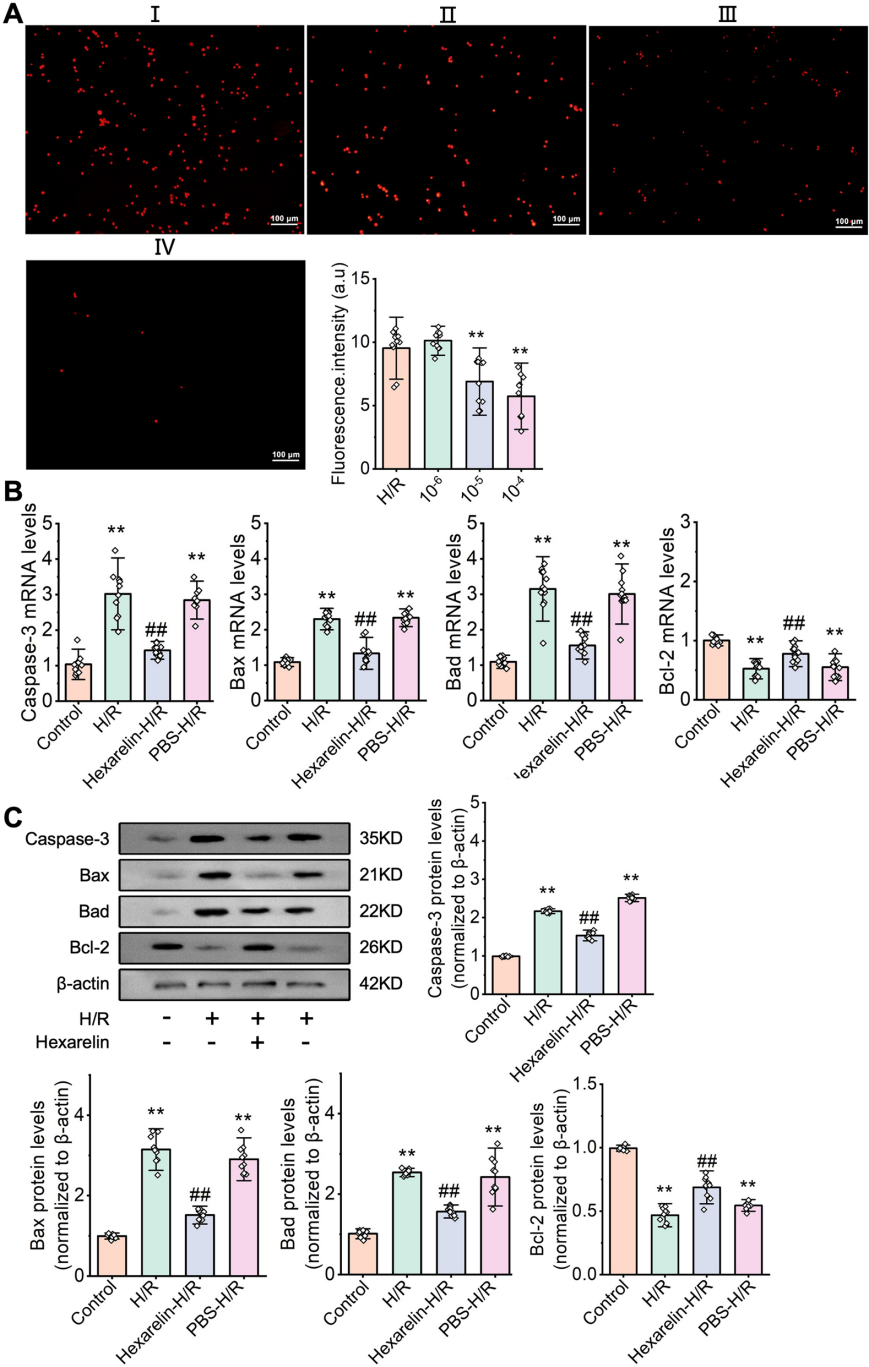
Hexarelin inhibited apoptosis in H/R-induced HK-2 cell. **A** Cell apoptosis of (I) H/R, pretreated with Hexarelin at a dose of (II)10^–6^, (III) 10^–5^ (IV) 10^–4^ were measured via PI staining; **B** relative fluorescent intensity of control, H/R, Hexarelin pretreatment before H/R and PBS pretreatment before H/R groups; **C**–**F** mRNA level of Caspase-3, Bax, Bad and Bcl-2 measured by qRT-PCR; **G**–**K** protein level of Caspase-3, Bax, Bad and Bcl-2 detected by Western blot analysis. *P < 0.05, **P < 0.01 versus control; ^#^*P* < 0.05, ^##^*P* < 0.01 versus AKI

Subsequently, we studied the Hexarelin on cell apoptosis induced by H/R-induced HK-2 cells. As depicted in Fig. 4A, Hexarelin significantly reduced the number of PI-positive cells post-H/R. Furthermore, we observed a remarkable upregulation of apoptosis-related proteins, e.g., Caspase-3, and downregulation of anti-apoptotic protein, e.g., Bcl-2, in post-H/R HK-2 cells. Pre-administration of Hexarelin reversed those tendencies (*P* < 0.05, Fig. 4B), which were further validated by western blot analysis (*P* < 0.05, Fig. 4C). Collectively, these findings indicated that Hexarelin exhibits an anti-apoptosis effect.

### Apoptosis regulatory effect of Hexarelin is associated with MDM2

**Fig. 5:**
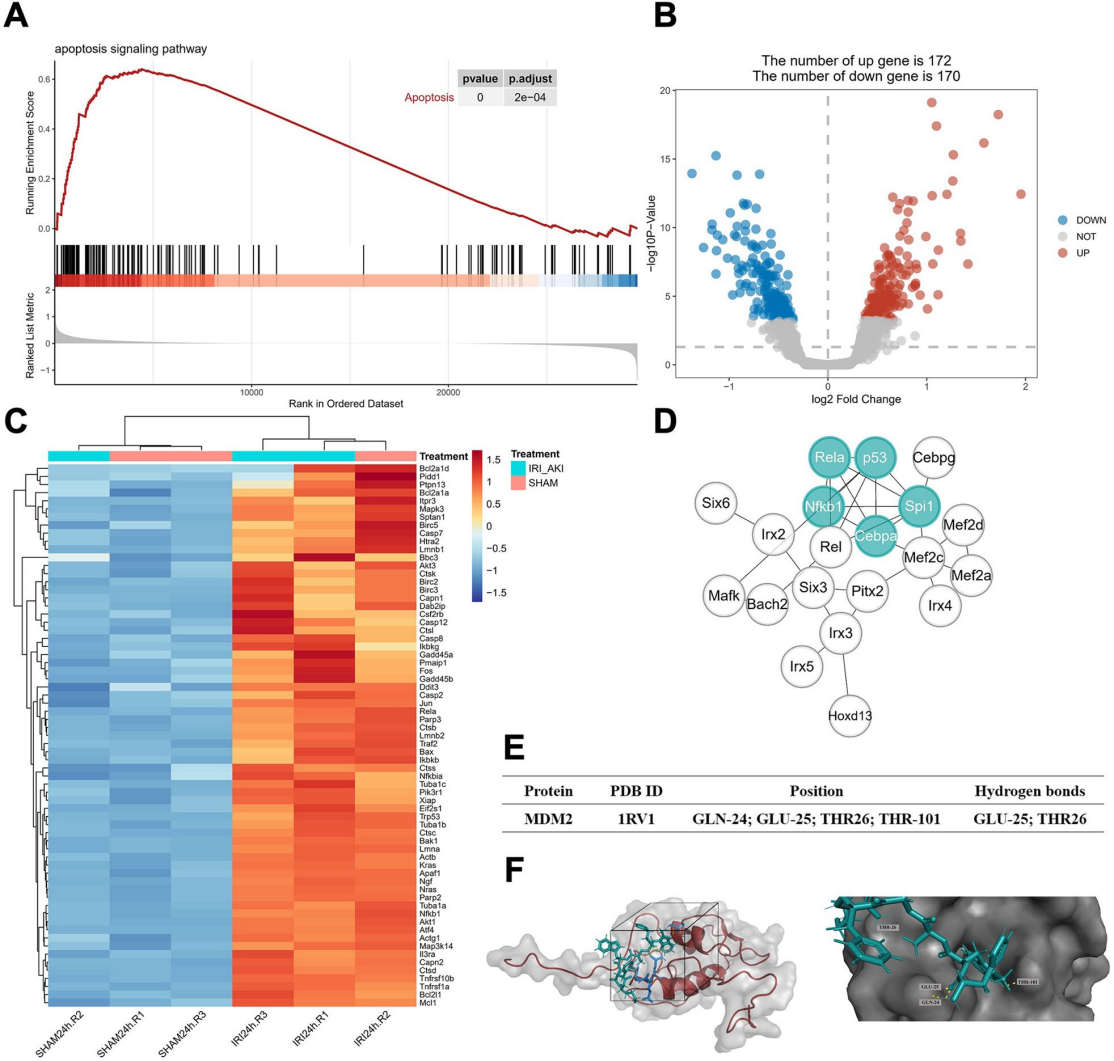
Hexarelin protected apoptosis against I/R-induced AKI associated with MDM2 and p53. **A** GSEA analysis of GEO dataset of GSE98622 samples; **B** Volcano plot DEGs of IR-AKI samples and (**C**) Heatmap of genes of apoptosis signaling pathway; **D** 5 core transcription factors were obtained using MCC algorithm after transcription factors enrichment prediction analysis; **E** Molecular docking was performed to confirm the combination of Hexarelin and MDM2; **F** Detailed combination information of Hexarelin and MDM2, the two formed hydrogen bonds at the position of GLU-25 and THR-26 from MDM2 on a sizeable hydrophobic pocket in the N-terminal domain of MDM2

A series of bioinformatics approaches were employed to study the underlying mechanism of Hexarelin (Fig. 5). As shown in Fig. 5B, there were 172 upregulated and 170 downregulated genes after AKI. GSEA revealed that 67 genes, including p53 and Bax, were enriched in the apoptosis signaling pathway, all of which were upregulated in I/R-induced AKI kidney (Fig. 5A, C, adj. *P* < 0.05). Furthermore, we investigated apoptosis-associated transcription factors in I/R-induced AKI. As a result, transcription factors with 50 motifs involved in the AKI (Additional file 2: Table S2, NES > 3), with five factors (p53, CEBP-α, NF-κB1, RelA, and Spi1) were identified as hub genes that regulate cell apoptosis (Fig. 5D). To further understand the pharmacological activity of Hexarelin, we performed both reverse docking and molecular docking. Consequently, there are 279 proteins were targets of Hexarelin, e.g., MDM2, a significant inhibitor of p53 (Additional file 3: Table S3, normalized fit score > 0.5). Moreover, molecular docking revealed a strong binding between Hexarelin and MDM2, with a binding energy of -8.26. Specifically, the two molecules formed hydrogen bonds at the position of GLU-25 and THR-26 in MDM2's sizeable hydrophobic pocket within the N-terminal domain, which serves as a core transcription site regulating p53. Collectively, these findings indicate that Hexarelin partially protects apoptosis in I/R-induced AKI through its targeting of MDM2 via the MDM2/p53 pathway.

### *Hexarelin therapeutic effects on AKI *via* targeting MDM2 through MDM2/p53 pathway*

**Fig. 6:**
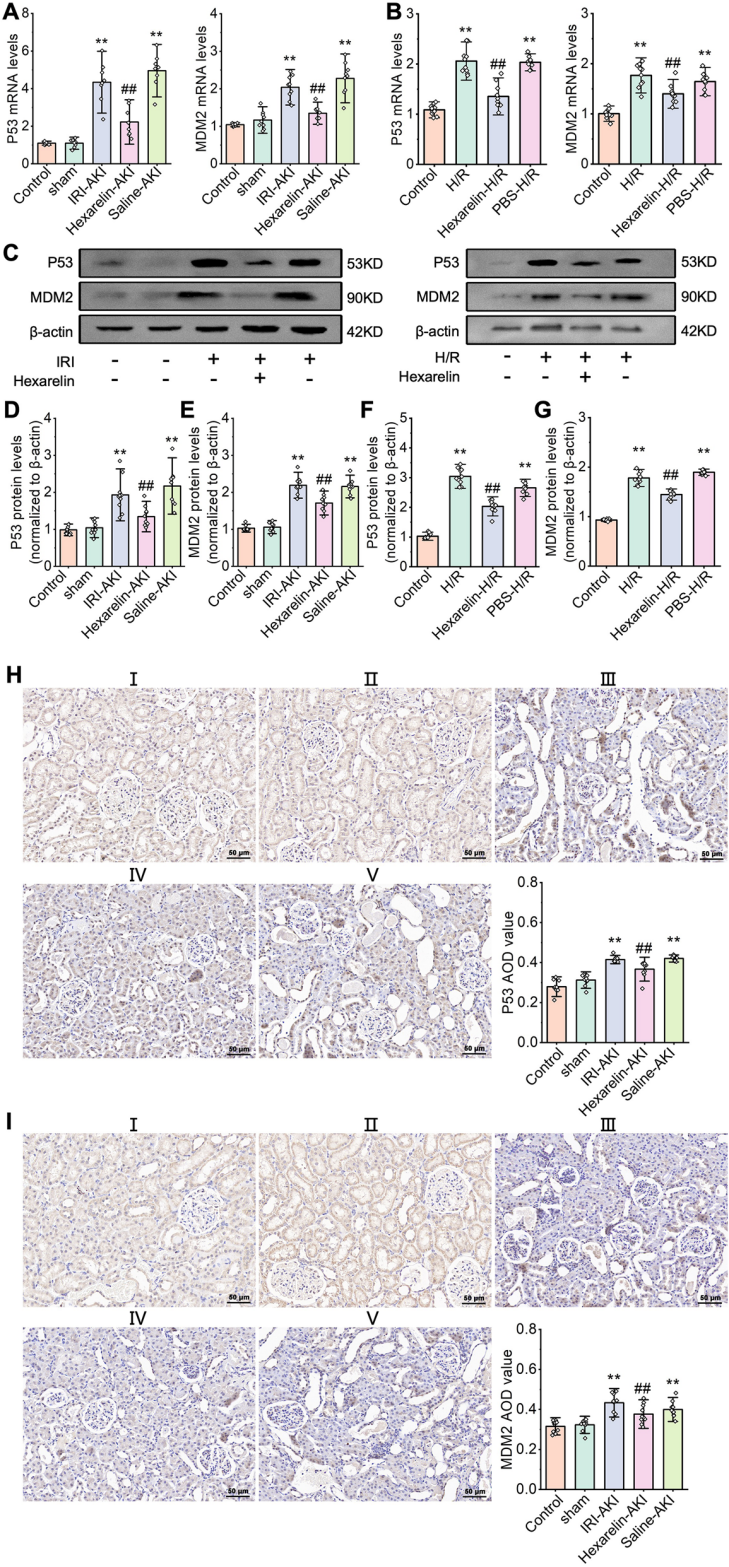
The expression of MDM2 and p53 after I/R-induced AKI and H/R exposure. mRNA levels of (**A**) p53 and (**B**) MDM2 detected by qRT-PCR; protein levels **C**–**G** Level of p53, MDM2 tested by western blot analysis; **H**–**I** Immunohistochemistry was used to detect the expression of p53, MDM2 in (I) control, (II) sham, (III) AKI, (IV) Hexarelin-AKI, and (V) Saline-AKI groups. *P < 0.05, **P < 0.01 versus control; ^#^*P* < 0.05, ^##^*P* < 0.01 versus AKI

To identify the relationship between Hexarelin and MDM2/p53, we assessed the expression levels of p53 and MDM2 both in vivo and in vitro. As a result, we observed a significant upregulation of p53 and MDM2 in the post-ischemic kidney, while decreasing in the Hexarelin-treated rat kidney. These findings suggest the involvement of p53 and MDM2 in the protective effects of Hexarelin against AKI (Fig. 6). Collectively, our results indicate that Hexarelin has the ability to inhibit cell apoptosis in I/R-induced AKI, and this effect is mediated by the interplay between MDM2 and p53.

## Discussion

**Fig. 7:**
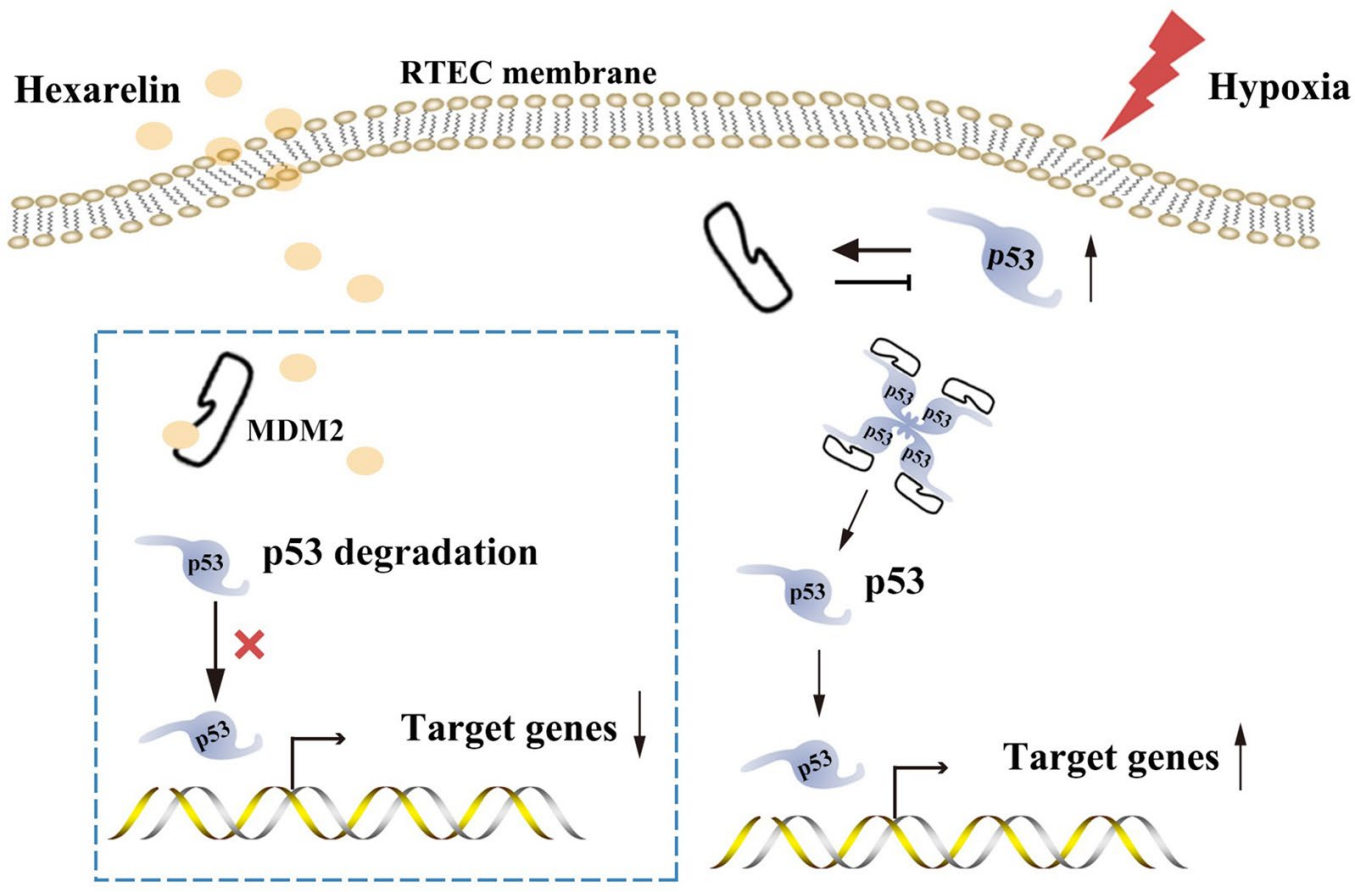
The potential mechanisms of Hexarelin protect against I/R-induced AKI. Hexarelin targeted MDM2 subsequently decreased p53, inhibiting apoptotic cell transcription promoter to protect cell apoptosis against I/R-induced AKI

AKI poses a significant risk for the development of CKD and its progression to end-stage renal disease, leading to complications and high mortality [24, 25]. Among the various causes of AKI, IRI is prevalent and contributes to the development of CKD. In this study, we identified p53 as a key transcription factor regulating cell apoptosis in I/R-induced AKI. Furthermore, we demonstrated, for the first time, that Hexarelin administration preserves renal function and mitigates cell apoptosis by interacting with MDM2 through the MDM2/p53 pathway, as confirmed by both in vivo and in vitro experiments. Mechanistically, through in vitro and in silico studies, we verified that Hexarelin targets MDM2, subsequently reducing p53 levels and inhibiting the transcriptional promotion of apoptotic cells, thereby safeguarding against apoptosis in I/R-induced AKI (Fig. 7). By uncovering the intricate molecular mechanism underlying Hexarelin's renal function improvement and protection against cell apoptosis in I/R-induced AKI, our study provides valuable insights.

IRI plays a significant role in the progression of AKI. Consequently, substances that inhibit IRI hold great potential in alleviating AKI, including both chemical agents [26–29] and natural-derived components [18, 30, 31]. While previous studies have reported the protective effects of Hexarelin on IRI in the heart and brain, potentially through its binding with CD36 or interaction with interleukin 1. However, there has been limited investigation into the protective effect of Hexarelin on the kidneys. Our research is the first to confirm this protective effect of Hexarelin on kidney tissue. Further, since growth hormone can also improve IRI, we detected its effects in our study. We found that administration of Hexarelin 7 days before surgery elicits no significant difference in this concentration of growth hormone compared with that of the AKI group (Additional file 4: Fig. S1), suggesting the protective effect of Hexarelin is irrelevant to promoting growth hormone secretion.

P53 plays a critical role in the cellular response to stress, including hypoxia [32]. Within the nucleus, p53 transcription activates a wide range of genes involved in apoptosis, while cytoplasmic p53 exerts its functions independent of transcription by directly interacting with cytoplasmic proteins, such as apoptotic effectors [33, 34]. Accumulating evidence supports the strong potential of p53 in preventing and treating AKI, as well as impeding the progression to CKD [35]. MDM2, a p53-specific E3 ubiquitin ligase, acts as the principal cellular antagonist of p53 and promotes cancer cell survival and growth through the degradation of the cell cycle regulator p53 [36]. Studies have shown that MDM2-mediated suppression of p53 is necessary for tubular regeneration during the healing phase of AKI [37]. Therefore, MDM2 represents a promising therapeutic target for alleviating I/R-induced AKI, and drugs targeting MDM2 that promote MDM2 expression and subsequently inhibit p53 may effectively ameliorate renal ischemia–reperfusion injury. In our in-silico analysis, we identified MDM2 as one of the targets of Hexarelin, leading to p53 inhibition and attenuation of I/R-induced AKI. Specifically, Hexarelin interacts with MDM2 through intermolecular hydrogen bonds at the side chain of GLU-25 and THR-26, which are positions known to stabilize the MDM2-p53 complex according to Kannan et al. [38]. These findings provide strong evidence that Hexarelin's interaction with MDM2, through the formation of intermolecular hydrogen bonds at GLU-25 and THR-26, facilitates p53 degradation and inhibits cell apoptosis by stabilizing the MDM2-p53 complex. However, it is important to note that the therapeutic use of Hexarelin in cancer may pose a risk of impaired (epithelial) healing, which partially aligns with this concept. Nevertheless, further research is needed to gather additional evidence.

## Materials and methods

### Animal experiment

Healthy male SPF SD rats (Jinan Pengyue Animal Centre) aged 8 weeks weighing 250–300 g were obtained from Jinan Pengyue Animal Centre. They were individually isolated in cages with controlled room temperature (21 ± 2 °C) and relative humidity (50 ± 15%). The rats were provided ad libitum access to food and water. A total of fifty rats were randomly assigned to 5 groups (*n* = 10), normal, sham, AKI, Hexarelin-AKI, and Saline-AKI. In the Hexarelin-AKI and Saline-AKI groups, intraperitoneal injections were performed 7 days prior to AKI surgery using either Hexarelin or saline. The selected Hexarelin dose was based on previous research, which demonstrated that it maximally stimulated growth hormone secretion and food intake without exhibiting drug toxicity within the range of concentrations 80–320 µg/kg [39].

The IRI-AKI model was established by inducing bilateral renal artery occlusion for 45 min followed by 24 h of reperfusion, as previously described [40]. The Sham group underwent the same surgical procedure as the AKI group, except that the renal pedicle was not clamped. Blood samples were collected from the inferior vena cava, tissues were collected and stored at − 80 °C until use.

### Renal function and histology

Serum creatinine and blood urea nitrogen (BUN) were measured using the picric acid method. Blood samples were collected in vacutainer serum collection tubes and allowed to stand at room temperature for 1 h to clot. After centrifugation at 3000 rpm for 15 min at 4 °C, the serum supernatant was transferred to a clean tube and stored at − 20 °C.

The histology of kidney tissues was evaluated through hematoxylin and eosin (H&E) and Periodic Acid-Schiff (PAS) staining. Kidney tissues were fixed in 4% paraformaldehyde at 4 °C for 24 h. Subsequently, the tissues were paraffin-embedded, sectioned at 4 µm thickness for staining, and examined using a light microscope. Renal tubular damage was indicated by assessing renal shedding of the brush border, tubular necrosis, exfoliation of the epithelial cells, cast deposition, and vascular congestion as previously described. Briefly, renal tissue lesions were classified from 0 to 5 according to the intensity of damages, 0: no damage, 1: less than 20% damage, 2: 21–40% damage, 3: 41–60% damage, 4: 61–80% damage, and 5: more than 81% damage [41, 42].

### Serum growth hormone assay

Assays Serum GH concentrations were determined using an immunoradiometric assay (Hybritech Tandem-R hGH Kit, Hybritech, Liege, Belgium) following manufacturer's instruction.

### Bioinformatics analysis

Raw data of the RNA-Seq dataset (GSE98622) containing 3 control and 3 AKI samples were downloaded and counts per million (CPM) were calculated. Differentially expressed genes (DEGs) analysis was conducted using the “edgeR” package in R software. The false discovery rate (FDR) was calculated using Benjamini & Hochberg method. log_2_FC > 1.5 and FDR < 0.05 were used as the threshold for DEGs. Then Gene-set enrichment analysis (GSEA) was performed for the dataset, with the normalized enrichment score (NES) and FDR serving as measures of enrichment magnitude and statistical significance, respectively [43]. To identify transcription factor binding motifs, the “*RcisTarget*” package was employed [44]. The Cystoscope plugin*, “CytoHubba”* was used to calculate node scores of genes using the Degree method, and the top 5 genes were selected as hub genes [45].

Hexarelin structure was downloaded from PubChem Project (https://pubchem.ncbi.nlm.nih.gov/). Reverse docking was conducted using the Pharmmapper Database server (http://lilab.ecust.edu.cn/pharmmapper/). The parameters were set as follows: all proteins set; normalized fit score ≥ 0.3 was considered a potential target. Molecular docking was performed using Autodock software (Version 4.2). Briefly, prior to docking, the ligands and proteins acted as receptors were firstly pretreated by removing solvent molecules and ligand, as well as hydrogenation and electron addition. The docking procedure utilized a Semi flexible docking approach with the Lamarckian genetic algorithm. The results were visualized using Pymol software (Version 4.6.0).

### Cell culture and hypoxia/reoxygenation (H/R) treatment

HK-2 cells were purchased from Shanghai Institute of Cell Biology (CAS, Shanghai, China). Cell culture and construction of the hypoxia/reoxygenation (H/R) model were based on previous studies used [41]. Briefly, HK-2 cells were cultured in DMEM-F12 medium containing 10% fetal bovine serum (Gibco, United States) and 100X penicillin–streptomycin solution (10 KU/m penicillin, 10 mg/ml streptomycin, P1410, Solarbio, China). The cells were incubated in a 37 °C humidified incubator in an atmosphere of 5% CO_2_. Then the medium was replaced with the glucose-free serum-free medium when cells were plated to 80% confluence before H/R treatment. H/R group cells were exposed to hypoxia including 5% CO_2_, 1% O_2_, and 94% N_2_ for different durations, followed by reoxygenation for 3 h. The control group was incubated at normoxic conditions without a medium change.

### Cell viability test

The cell viability test was assessed using a previously established method [46]. The CCK-8 kit was employed to measure cell viability, following the instructions provided by the manufacturer of the kit. The absorbance value was measured at 450 nm.

### Quantitative real-time PCR (qRT-PCR)

Total RNA from kidney tissue was isolated by the Trizol method, and cDNA synthesis was performed using the PrimeScript RT Reagent Kit with gDNA Eraser (RR047A, Takara, Japan). For animal samples, 1 mg of RNA was used, while for cell samples, 0.5 mg of RNA was used. Then Quantitative Real-Time PCR (qRT-PCR) was performed by an ABI-7500 instrument with TB Green Premix Ex Taq II (RR820A, Takara, Japan). The reaction conditions were as follows: 95 °C 30 s × 1 cycle; 95 °C 5 s, 60 °C 40 s × 40 cycles, and data were normalized by β-actin. The 2^−ΔΔCT^ method was used to calculate the relative expression of mRNA. Primer sequences used in the experiments are shown in Additional file 1: Table S1.

### Western blot analysis

Western blotting analysis was conducted following established protocols [47]. Protein samples were collected using Standard RIPA buffer with PMSF and phosphatase inhibitor cocktail (1000:10:1 ratio) and centrifugated at 4 × for 15 min at 12000 rpm. The protein concentrations were determined using the BCA Protein Assay Kit. The protein supernatants were mixed with 5 × SDS loading buffer and heated at 95 °C for 15 min. A total of 30 µg of protein mixture solution was loaded onto a 10% SDS-PAGE and transferred onto 0.2 μm PVDF membranes (Millipore, United States). The membrane was blocked with 5% skimmed milk at room temperature for 1 h. Subsequently, the membranes were incubated overnight at 4 °C with primary antibodies, including β-actin (E-AB-20058, Elabscience Biotechnology), Caspase-3 (1:1000, Abcam), BCL2-associated X apoptosis regulator (Bax, 1:1000, Elabscience Biotechnology), BCL2 apoptosis regulator (Bcl-2, 1:1000, Elabscience Biotechnology), BCL2-associated agonist of cell death (BAD, AF7927, Affinity), P53 (1:3000, Elabscience Biotechnology), murine double minute2 (MDM2,1:1000, Elabscience Biotechnology) diluted with PBST at 4 °C overnight. After washing with PBST, the membranes were incubated with secondary antibodies (goat anti-rabbit or goat anti-mouse IgG-HRP (1:10000, Absin, China) at room temperature for 1 h. The protein bands were visualized using an excellent chemiluminescent substrate detection kit (Elabscience Biotechnology) and target bands were subjected to grayscale analysis using ImageJ software (Version 1.52a).

### Immunohistochemistry

Expression of MDM2 and P53 waas assessed using immunohistochemistry. Briefly, the tissue sections were incubated with a blocking buffer containing 3% BSA (Servicebio) for 1 h. Subsequently, the sections were incubated overnight at 4 °C with primary antibodies, including anti-MDM2 antibody (1:200, Elabscience Biotechnology) and anti-P53 antibody (1:200, Elabscience Biotechnology) incubated overnight at 4 °C. After washing, the sections were incubated with secondary antibodies (Elabscience Biotechnology) for 50 min at room temperature. DAB Kit (G1211, Servicebio) was used for staining. A light microscope was used for morphology assessment. Average optical density (AOD, AOD = integrated density/area) MDM2 and P53 were analyzed using Image J software.

### TdT-mediated dUTP nick-end labeling (TUNEL)

TUNEL assay was performed to assess the apoptotic cells using TUNEL Apoptosis Detection Kit (Alexa Fluor 488, CA) following the manufacturer’s instructions. Briefly, the HK-2 cells were washed with PBS after H/R and fixed with 4% Paraformaldehyde. Subsequently, the cells were stained and examined using a fluorescence microscope.

### Propidium iodide (PI) staining

Cells at 60% confluence were fixed with 4% paraformaldehyde for 1–2 h at 4 °C. Subsequently, the cells were stained with PI (Yeasonbiotech, China) while being protected from light, at 4 °C for 30 min. Apoptotic cells, stained red by PI, were observed using a fluorescence microscope at 535 nm. The characteristic morphological changes of apoptotic cells were observed: nuclear chromatin gathers on one side of the nuclear membrane, showing a crescent shape; the nucleus of late apoptotic cells fragments into prototype bodies of varying sizes, which were surrounded by cell membranes.

### Statistical analysis

All data analysis was performed using OriginPro software (version 2021b, OriginLab, Northampton, MA, USA). Data were checked for normal distribution before every statistical analysis. For multiple comparisons, continuous variables were subjected to one-way analysis of variance followed by the Bonferroni post hoc test. All data are shown as mean ± SD. *P* < 0.05 was considered to indicate statistical significance.

## Conclusions

In conclusion, we provide novel evidence that Hexarelin inhibits cell apoptosis in I/R-induced AKI via targeting MDM2 by forming hydron bonds at GLU-25 and THR-26 from MDM2 and then promotes the degradation of p53. These findings suggest that Hexarelin may be an efficacy therapeutic medicine for I/R-induced AKI.

### Supplementary Information

**Additional file 1: Table S1.** Genes and primers used in the experiments.

**Additional file 2: Table S2**. Potential transcription factors involved in apoptosis in the I/R-induced AKI.

**Additional file 3: Table S3.** Potential targets of Hexarelin.

**Additional file 4:**Concentration of growth hormone after admnistration of Hexarelin for 7 days.

## Data Availability

The RNA-Seq gene expression data have been publicly available in a GEO repository (GSE98622).
