# Supplementary material for: Hexarelin alleviates apoptosis on ischemic acute kidney injury via MDM2/p53 pathway
Source: Eur J Med Res. 2023 Sep 14;28:344. doi: 10.1186/s40001-023-01318-w (PMC10500723; doi:10.1186/s40001-023-01318-w)
Supplement: Supplementary file 1 — Additional file 1: Table S1. Genes and primers used in the experiments. [file 40001_2023_1318_MOESM1_ESM.docx]

**Additional file Table S1. Genes and primers used in the experiments**

| Genes | Forward | Reverse |
| --- | --- | --- |
| r-KIM-1 | TCCACACATGTACCAACATCAA | GTCACAGTGCCATTCCAGTC |
| r-caspase-3 | GAGGAGATGGCTTGCCAGAA | CTTGTGCGCGTACAGCTTCA |
| r-Bad | TGAGGAAGATGAAGGGATGGA | CGAGGAAGTCCCTTGAAGGAA |
| r-Bax | ATGTTTTCTGACGGCAACTTC | AGTCCAATGTCCAGCCCAT |
| r-Bcl2 | CAGATGCACCTGACGCCCTT | CCCAGCCTCCGTTATCCTGGA |
| r-p53 | ACAGCGTGGTGGTACCTTATGA | GGTTCCCACTGGAGTCTTCCA |
| r-MDM2 | GCGAGCGGAGACGGACACAC | GGGCTCTGTGGCGCTTCCTC |
| r-β-actin | CCTGGACTTCGAGCAAGAGATG | AGGAAGGAAGGCTGGAAGAGTG |
| h-HIF1-α | ACCGCTGAAACGCCAAAG | TCCATCGGAAGGACTAGGTGTCT |
| h-caspase-3 | TGCATACTCCACAGCACCTGGTTA | CATGGCACAAAGCGACTGGATGAA |
| h-Bad | GGAGGATGAGTGACGAGTTTGTG | ATCCCACCAGGACTGGAAGAC |
| h-Bax | TGGCAGCTGACATGTTTTCTG | TCCCGGAGGAAGTCCAATG |
| h-Bcl2 | ACGGTGGTGGAGGAGCTCTT | GCCGGTTCAGGTACTCAGTCAT |
| h-p53 | CCTGAGGTTGGCTCTGACTGTA | CACGCACCTCAAAGCTGTTC |
| h-MDM2 | TGTGAAGGAGCACAGGAAAA | TCCTTCAGATCACTCCCACC |
| h-β-actin | GGGAAATCGTGCGTGACATT | GGAACCGCTCATTGCCAAT |

^#^ r: rats; h: human
