# Supplementary material for: Hexarelin alleviates apoptosis on ischemic acute kidney injury via MDM2/p53 pathway
Source: Eur J Med Res. 2023 Sep 14;28:344. doi: 10.1186/s40001-023-01318-w (PMC10500723; doi:10.1186/s40001-023-01318-w)
Supplement: Supplementary file 2 — Additional file 2: Table S2. Potential transcription factors involved in apoptosis in the I/R-induced AKI. [file 40001_2023_1318_MOESM2_ESM.docx]

**Additional file Table S2. Potential transcription factors involved in apoptosis in the I/R-induced AKI**

| Motif | NES | AUC | TF-highConf |
| --- | --- | --- | --- |
| hocomoco__SPI1_MOUSE.H11MO.0.A | 5.42 | 0.111 | Spi1 |
| cisbp__M1907 | 4.93 | 0.103 | Spi1 |
| transfac_pro__M07224 | 4.92 | 0.103 | Spi1 |
| homer__AGAGGAAGTG_PU.1 | 4.88 | 0.102 | Spi1 |
| transfac_pro__M02114 | 4.82 | 0.101 | Pitx2 |
| hocomoco__MEF2C_MOUSE.H11MO.0.A | 4.59 | 0.097 | Mef2c |
| hocomoco__MEF2D_MOUSE.H11MO.0.A | 4.26 | 0.092 | Mef2d |
| cisbp__M0986 | 4.19 | 0.091 | Irx3 |
| cisbp__M6032 | 4.05 | 0.089 | Hoxd13 |
| cisbp__M6105 | 3.93 | 0.087 | Trp53 |
| transfac_pro__M08825 | 3.92 | 0.087 | Nfib |
| transfac_pro__M00482 | 3.88 | 0.086 | Pitx2 |
| transfac_public__M00232 | 3.83 | 0.085 | Mef2a |
| taipale__Hoxd13_DBD_NCTCRTAAAAN | 3.82 | 0.085 | Hoxd13 |
| jaspar__MA0591.1 | 3.77 | 0.084 | Mafk |
| cisbp__M0988 | 3.69 | 0.083 | Irx6 |
| cisbp__M0942 | 3.57 | 0.081 | Irx4 |
| cisbp__M6122 | 3.52 | 0.080 | Spi1 |
| hocomoco__MEF2A_MOUSE.H11MO.0.A | 3.52 | 0.080 | Mef2a |
| transfac_pro__M02896 | 3.51 | 0.080 | Spi1 |
| transfac_public__M00054 | 3.5 | 0.080 | Rel; Rela |
| cisbp__M0313 | 3.46 | 0.079 | Cebpg |
| cisbp__M6106 | 3.46 | 0.079 | Trp73 |
| transfac_public__M00052 | 3.45 | 0.079 | Rela |
| hocomoco__BACH2_MOUSE.H11MO.0.A | 3.45 | 0.079 | Bach2 |
| cisbp__M0987 | 3.44 | 0.079 | Irx5 |
| transfac_pro__M03545 | 3.43 | 0.079 | Rel |
| cisbp__M0316 | 3.38 | 0.078 | Nfil3 |
| hocomoco__TF65_MOUSE.H11MO.0.A | 3.3 | 0.077 | Rela |
| transfac_public__M00053 | 3.3 | 0.077 | Rel |
| transfac_pro__M01203 | 3.3 | 0.077 | Spi1 |
| transfac_public__M00026 | 3.26 | 0.076 | Mef2a |
| cisbp__M0909 | 3.24 | 0.076 | Irx2 |
| cisbp__M0293 | 3.21 | 0.075 | Tef |
| cisbp__M1044 | 3.21 | 0.075 | Hoxc12 |
| cisbp__M1016 | 3.2 | 0.075 | Six3 |
| cisbp__M6137 | 3.11 | 0.073 | Rela |
| cisbp__M6047 | 3.11 | 0.073 | Mafb |
| transfac_pro__M07056 | 3.09 | 0.073 | Pitx2 |
| homer__GGAAATTCCC_NFkB-p65-Rel | 3.09 | 0.073 | Rela |
| cisbp__M0312 | 3.08 | 0.073 | Junb |
| cisbp__M0304 | 3.08 | 0.073 | Cebpa |
| cisbp__M0937 | 3.08 | 0.073 | Six6 |
| transfac_pro__M03551 | 3.06 | 0.073 | Junb |
| cisbp__M1963 | 3.05 | 0.072 | Zfx |
| jaspar__MA0146.2 | 3.02 | 0.072 | Zfx |
