# Supplementary material for: Hexarelin alleviates apoptosis on ischemic acute kidney injury via MDM2/p53 pathway
Source: Eur J Med Res. 2023 Sep 14;28:344. doi: 10.1186/s40001-023-01318-w (PMC10500723; doi:10.1186/s40001-023-01318-w)
Supplement: Supplementary file 3 — Additional file 3: Table S3. Potential targets of Hexarelin. [file 40001_2023_1318_MOESM3_ESM.docx]

**Additional file Table S3. Potential targets of Hexarelin**

| **PDB_ID** | **Norm Fit** | **UniProtKB** | **gene name** | **PDB_ID** | **Norm Fit** | **UniProtKB** | **gene name** |
| --- | --- | --- | --- | --- | --- | --- | --- |
| 1w7h | 0.989 | Q16539 | MAPK14 | 1le6 | 0.678 | O15496 | PLA2G10 |
| 1w8m | 0.972 | P62937 | PPIA | 1yk7 | 0.678 | CATK_HUMAN | CTSK |
| 1xkb | 0.969 | FA10_HUMAN | F10 | 3cs8 | 0.677 | PPARG_HUMAN | PPARG |
| 1if4 | 0.960 | CAH2_HUMAN | CA2 | 1hx1 | 0.673 | BAG1_HUMAN | BAG1 |
| 1eub | 0.955 | MMP13_HUMAN | MMP13 | 1ovz | 0.670 | FCAR_HUMAN | FCAR |
| 1oja | 0.947 | AOFB_HUMAN | MAOB | 1nqc | 0.668 | CATS_HUMAN | CTSS |
| 1dvz | 0.944 | TTHY_HUMAN | TTR | 1ld8 | 0.668 | FNTA_HUMAN | FNTA |
| 1ogs | 0.935 | GLCM_HUMAN | GBA | 1gcz | 0.667 | MIF_HUMAN | MIF |
| 1z68 | 0.918 | SEPR_HUMAN | FAP | 2piu | 0.667 | ANDR_HUMAN | AR |
| 2afu | 0.912 | QPCT_HUMAN | QPCT | 2jdt | 0.665 | P00517 | PRKACA |
| 2vta | 0.912 | P24941 | CDK2 | 1p4f | 0.661 | DAPK1_HUMAN | DAPK1 |
| 1cb0 | 0.905 | Q13126 | MTAP | 1juj | 0.658 | TYSY_HUMAN | TYMS |
| 1xlv | 0.898 | CHLE_HUMAN | BCHE | 1zxc | 0.653 | ADA17_HUMAN | ADAM17 |
| 1unh | 0.890 | CD5R1_HUMAN | CDK5R1 | 2f6y | 0.652 | PTN1_HUMAN | PTPN1 |
| 1fh0 | 0.884 | CATL2_HUMAN | CTSV | 2ohq | 0.649 | BACE1_HUMAN | BACE1 |
| 2brg | 0.879 | CHK1_HUMAN | CHEK1 | 1mx9 | 0.643 | EST1_HUMAN | CES1 |
| 1xn0 | 0.877 | PDE4B_HUMAN | PDE4B | 1kbo | 0.643 | NQO1_HUMAN | NQO1 |
| 2hck | 0.877 | HCK_HUMAN | HCK | 2p2h | 0.642 | VGFR2_HUMAN | KDR |
| 4pah | 0.861 | PH4H_HUMAN | PAH | 1u3v | 0.638 | ADH1B_HUMAN | ADH1B |
| 1jdn | 0.850 | ANPRC_HUMAN | NPR3 | 1tbb | 0.638 | PDE4D_HUMAN | PDE4D |
| 1a28 | 0.731 | PRGR_HUMAN | PGR | 1azm | 0.637 | CAH1_HUMAN | CA1 |
| 1hak | 0.727 | ANXA5_HUMAN | ANXA5 | 1ry8 | 0.636 | AK1C3_HUMAN | AKR1C3 |
| 1j78 | 0.718 | VTDB_HUMAN | GC | 1so2 | 0.634 | PDE3B_HUMAN | PDE3B |
| 1gpz | 0.716 | P00736 | C1R | 1a7a | 0.629 | SAHH_HUMAN | AHCY |
| 1shl | 0.712 | CASP7_HUMAN | CASP7 | 1ih0 | 0.625 | P63316 | TNNC1 |
| 1m4u | 0.711 | BMP7_HUMAN | BMP7 | 1uou | 0.623 | TYPH_HUMAN | TYMP |
| 1boz | 0.711 | DYR_HUMAN | DHFR | 2gv6 | 0.622 | ST14_HUMAN | ST14 |
| 1rsz | 0.707 | PNPH_HUMAN | PNP | 1mmq | 0.622 | MMP7_HUMAN | MMP7 |
| 1az1 | 0.702 | ALDR_HUMAN | AKR1B1 | 1q11 | 0.621 | P54577 | YARS1 |
| 1vyw | 0.701 | CCNA2_HUMAN | CCNA2 | 1n83 | 0.620 | RORA_HUMAN | RORA |
| 1w7m | 0.698 | KAT1_HUMAN | KYAT1 | 1u81 | 0.619 | P84077 | ARF1 |
| 1csb | 0.697 | CATB_HUMAN | CTSB | 1lhw | 0.619 | SHBG_HUMAN | SHBG |
| 1d7x | 0.695 | MMP3_HUMAN | MMP3 | 2byi | 0.619 | HS90A_HUMAN | HSP90AA1 |
| 1pmu | 0.693 | MK10_HUMAN | MAPK10 | 2pe2 | 0.618 | PDPK1_HUMAN | PDPK1 |
| 1agw | 0.690 | FGFR1_HUMAN | FGFR1 | 2ity | 0.617 | EGFR_HUMAN | EGFR |
| 1mfu | 0.689 | P04745 | AMY1A | 2hu6 | 0.616 | MMP12_HUMAN | MMP12 |
| 1o4f | 0.689 | SRC_HUMAN | SRC | 1qdd | 0.610 | P05451 | REG1A |
| 2fky | 0.687 | KIF11_HUMAN | KIF11 | 1a27 | 0.609 | P14061 | HSD17B1 |
| 2g1y | 0.687 | RENI_HUMAN | REN | 3h30 | 0.609 | P68400 | CSNK2A1 |
| 1fa9 | 0.682 | P06737 | PYGL | 2of4 | 0.608 | LCK_HUMAN | LCK |
| 2c47 | 0.681 | KC1G2_HUMAN | CSNK1G2 | 1vj5 | 0.605 | HYES_HUMAN | EPHX2 |
| 1w6j | 0.604 | ERG7_HUMAN | LSS | 1e8z | 0.558 | PK3CG_HUMAN | PIK3CG |
| 1t7v | 0.602 | ZA2G_HUMAN | AZGP1 | 2fb8 | 0.556 | BRAF_HUMAN | BRAF |
| 2rku | 0.600 | p53350 | PLK1 | 1isg | 0.555 | BST1_HUMAN | BST1 |
| 1wok | 0.596 | P09874 | PARP1 | 1jqh | 0.550 | IGF1R_HUMAN | IGF1R |
| 2oqi | 0.593 | DPP4_HUMAN | DPP4 | 2acl | 0.549 | RXRA_HUMAN | RXRA |
| 1e7i | 0.591 | ALBU_HUMAN | ALB | 2shp | 0.548 | PTN11_HUMAN | PTPN11 |
| 1a5h | 0.590 | TPA_HUMAN | PLAT | 1now | 0.546 | P07686 | HEXB |
| 1oec | 0.590 | P21802 | FGFR2 | 1m6w | 0.545 | ADHX_HUMAN | ADH5 |
| 1yxu | 0.588 | PIM1_HUMAN | PIM1 | 1mfv | 0.545 | P04745 | AMY1A |
| 1dic | 0.586 | CFAD_HUMAN | CFD | 1d3h | 0.543 | PYRD_HUMAN | DHODH |
| 1hkc | 0.581 | HXK1_HUMAN | HK1 | 1nd5 | 0.542 | PPAP_HUMAN | ACP3 |
| 1w22 | 0.581 | HDAC8_HUMAN | HDAC8 | 1nup | 0.537 | Q96T66 | NMNAT3 |
| 3eml | 0.580 | P29274 | ADORA2A | 1he2 | 0.534 | P30043 | BLVRB |
| 1mq0 | 0.579 | CDD_HUMAN | CDA | 2q6c | 0.533 | HMDH_HUMAN | HMGCR |
| 1k0n | 0.579 | O00299 | CLIC1 | 1og5 | 0.533 | CP2C9_HUMAN | CYP2C9 |
| 1dcy | 0.578 | P14555 | PLA2G2A | 1ctr | 0.530 | CALM1_HUMAN | CALM1 |
| 1of7 | 0.577 | ALDH2_HUMAN | ALDH2 | 1fe0 | 0.530 | O00244 | ATOX1 |
| 1ov4 | 0.576 | Q06520 | SULT2A1 | 2opy | 0.528 | XIAP_HUMAN | XIAP |
| 1wlj | 0.573 | ISG20_HUMAN | ISG20 | 3bzu | 0.525 | DHI1_HUMAN | HSD11B1 |
| 1wda | 0.573 | PADI4_HUMAN | PADI4 | 1xo2 | 0.525 | CDK6_HUMAN | CDK6 |
| 2vwv | 0.573 | EPHB4_HUMAN | EPHB4 | 1m6d | 0.524 | Q9UBX1 | CTSF |
| 1xbc | 0.570 | KSYK_HUMAN | SYK | 1r5h | 0.523 | MAP2_HUMAN | MAP2 |
| 1cgh | 0.570 | CATG_HUMAN | CTSG | 1imb | 0.520 | IMPA1_HUMAN | IMPA1 |
| 1wma | 0.568 | CBR1_HUMAN | CBR1 | 3dy6 | 0.520 | Q03181 | PPARD |
| 1w8b | 0.567 | FA7_HUMAN | F7 | 2i0g | 0.518 | ESR2_HUMAN | ESR2 |
| 1u4d | 0.567 | ACK1_HUMAN | TNK2 | 1o5f | 0.517 | P05981 | HPN |
| 5gal | 0.566 | LEG7_HUMAN | LGALS7 | 3cqw | 0.517 | AKT1_HUMAN | AKT1 |
| 1pl6 | 0.564 | DHSO_HUMAN | SORD | 1p5z | 0.516 | DCK_HUMAN | DCK |
| 1xan | 0.561 | P00390 | GSR | 3ewj | 0.510 | P78536 | ADAM17 |
| 1fo2 | 0.560 | Q9UKM7 | MAN1B1 | 1rv1 | 0.509 | **MDM2_HUMAN** | **MDM2** |
| 1t84 | 0.560 | WASP_HUMAN | WAS | 1iz2 | 0.507 | A1AT_HUMAN | SERPINA1 |
| 1xdc | 0.559 | P04179 | SOD2 | 2f57 | 0.507 | PAK5_HUMAN | PAK5 |
