# Supplementary figures and images for: Hexarelin alleviates apoptosis on ischemic acute kidney injury via MDM2/p53 pathway
Source: Eur J Med Res. 2023 Sep 14;28:344. doi: 10.1186/s40001-023-01318-w (PMC10500723; doi:10.1186/s40001-023-01318-w)

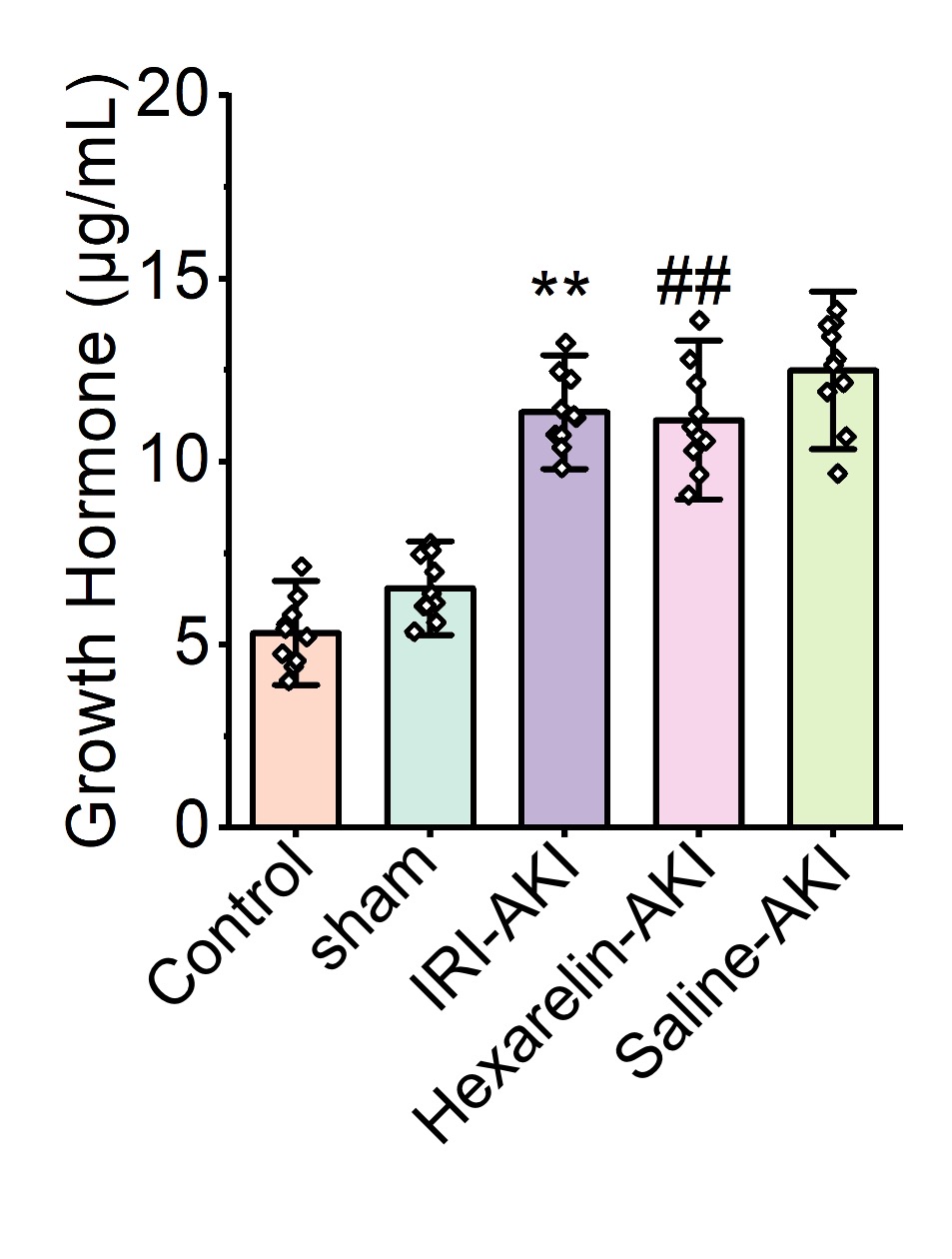

Supplement: Supplementary file 4 — Additional file 4:Concentration of growth hormone after admnistration of Hexarelin for 7 days. [file 40001_2023_1318_MOESM4_ESM.jpg]
